# Evaluating sleep quality and daytime sleepiness in nursing students: psychometric validation of the Pittsburgh Sleep Quality Index and the Epworth Sleepiness Scale

**DOI:** 10.1186/s12912-025-04044-2

**Published:** 2025-11-25

**Authors:** Carlos Alberto Henao Periañez, Cruz Deicy Jaramillo-Bolívar, Marcio Alexander Castillo-Díaz

**Affiliations:** 1 Facultad de Salud, Escuela de Enfermería, Universidad del Valle, Cali, Colombia; 2 Facultad de Ciencias de la Salud, Programa de Enfermería, Universidad Libre seccional Cali, Cali, Colombia; 3 Facultad de Ciencias Sociales, Departamento de Psicología/Maestría en Psicometría y Evaluación Educativa, Universidad Nacional Autónoma de Honduras, Tegucigalpa, Honduras

**Keywords:** Sleep, Sleep wake disorders, Nursing students, Structural validity, PSQI, ESS

## Abstract

**Background:**

Sleep problems are common among nursing students, yet the psychometric performance of widely used measures remains underexamined. The Pittsburgh Sleep Quality Index (PSQI) and the Epworth Sleepiness Scale (ESS) are frequently applied to assess sleep quality and daytime sleepiness, respectively, but evidence of their validity and reliability in nursing-student populations is limited.

**Aims:**

This study aimed to evaluate the structural validity, reliability, and convergent validity of the PSQI and ESS in a sample of nursing students.

**Methods:**

This methodological study was conducted among 368 nursing students enrolled at a university in Cali, Colombia. The Spanish versions of the PSQI and ESS were administered. In a subsample of 87 participants, a second assessment was conducted approximately eight weeks later to examine temporal stability. Item-level confirmatory factor analyses were conducted, internal consistency was assessed, latent variable correlations were examined via structural equation modeling, and test–retest reliability was evaluated using intraclass correlation coefficients (ICC).

**Results:**

The theoretical unidimensional structure of the PSQI demonstrated poor model fit (CFI = 0.919; RMSEA = 0.137). In contrast, a respecified unidimensional model that included two residual correlations yielded substantially improved fit indices (CFI = 0.980; RMSEA = 0.065). For the ESS, a unidimensional model incorporating one residual correlation showed an acceptable fit (CFI = 0.997; RMSEA = 0.057). Both instruments exhibited satisfactory internal consistency, as evidenced by composite reliability (CR) coefficients of 0.774 and 0.904 for the PSQI and ESS, respectively. Their temporal stability was fair to moderate, as indicated by ICC of 0.464 for the PSQI and 0.567 for the ESS. Additionally, the structural model indicated a statistically significant correlation between the two scales (*r* = .721, *p* < .001).

**Conclusions:**

The PSQI and ESS demonstrate adequate psychometric properties for assessing sleep quality and daytime sleepiness in nursing students. These findings support the use of both instruments mainly in nursing-student populations and comparable settings, helping to provide a more comprehensive evaluation of sleep health.

**Supplementary Information:**

The online version contains supplementary material available at 10.1186/s12912-025-04044-2.

## Statement of Significance

Sleep problems are common among nursing students, yet commonly used instruments are seldom validated in this population. This study is the first in Colombia to evaluate the psychometric performance of the Pittsburgh Sleep Quality Index (PSQI) and the Epworth Sleepiness Scale (ESS) among nursing students. Using confirmatory factor analysis and test–retest reliability assessment, we show that both instruments demonstrate acceptable structure, internal consistency, temporal stability, and convergent validity. These results support the use of the PSQI and ESS for screening and monitoring sleep health in academic and clinical training settings, enabling more dependable surveillance, targeted supports, and evaluation of interventions that may improve student well-being and patient safety.

**Supplementary Information:**

The online version contains supplementary material available at 10.1186/s12912-025-04044-2.

## Introduction

Sleep is an essential physiological process for human physical, mental, and emotional health [1]. Sleep quality is defined as an individual’s overall satisfaction with various aspects of the sleep experience [2]. Good sleep quality is associated with enhanced emotion regulation, improved concentration, and better cognitive performance [3, 4]. Conversely, sleep disturbances may have detrimental effects on overall health, increasing the risk of both physical and mental disorders, as well as the likelihood of occupational and traffic accidents [5–8].

University students show a high prevalence of sleep disturbances, which are associated with elevated academic stress and symptoms of anxiety and depression, negatively impacting their daily functioning and mental health throughout their academic training [9]. Within this population, nursing students face distinct challenges, including demanding academic workloads, emotional strain during clinical placements, and night shifts while studying—factors that can significantly compromise their sleep quality [10, 11]. Moreover, high levels of maladaptive coping strategies have been reported in this population, often manifested through symptoms of anxiety, depression, and sleep disturbances [12].

The consequences of poor sleep quality for nursing students are relevant, as they can impair both their academic performance [13, 14] and their clinical performance [15], and even compromise their competence in patient safety during professional practices [16]. In this context, the availability of valid and reliable instruments to assess sleep quality among nursing students is essential for the early identification of sleep disturbances and for informing the development of preventive strategies [17]. Although several instruments have been developed to assess various dimensions of sleep and its impact on daily functioning [18], there is a lack of empirical evidence evaluating the psychometric properties of health-related sleep measures specifically in nursing student populations.

One of the most widely used instruments worldwide is the Pittsburgh Sleep Quality Index (PSQI), a self-report questionnaire designed to assess sleep quality over the past month [19]. The PSQI comprises seven components: subjective sleep quality, sleep latency, sleep duration, habitual sleep efficiency, sleep disturbances, use of sleep medication, and daytime dysfunction [19]. Its extensive use in clinical and research settings is attributable to its ease of administration and the evidence supporting its sound psychometric properties [18]. The PSQI has been translated and validated in numerous languages, including English [20], Italian [21], Arabic [22], Portuguese [23, 24] and Spanish [25, 26], among others. The psychometric properties of the PSQI have been evaluated across diverse populations, including individuals with cancer [22, 27, 28], fibromyalgia [25], pregnant women [20, 26], older adults [23], children [21], adolescents and young adults [29], and university students [30–32].

The Epworth Sleepiness Scale (ESS), on the other hand, is a widely used instrument in clinical and epidemiological research for assessing daytime sleepiness in everyday situations [33, 34]. The ESS assesses an individual’s typical likelihood of dozing off in various situations, each selected to represent the spectrum of sleepiness commonly experienced in daily life [33]. The ESS has been translated and validated in multiple languages, including English [35, 36], Portuguese [37, 38], German [39], Japanese [40] and Spanish [41–43], among others. Its use has been documented in individuals with obstructive sleep apnea [42], epilepsy [35], and Parkinson’s disease [44], as well as in older adults [36, 39], children and adolescents [38], and university students [43, 45].

Despite the widespread use of the PSQI and ESS to assess sleep quality and daytime sleepiness, systematic reviews and meta-analyses have identified notable limitations in their psychometric properties [34, 46–48]. The PSQI has shown good internal consistency and overall utility as a screening tool; however, inconsistencies in its factor structure and in the interpretation of its global score have been reported, raising concerns about its ability to discriminate effectively between good and poor sleepers across diverse populations [46, 48]. On the other hand, while the ESS has demonstrated acceptable internal consistency, findings from a recent meta-analysis revealed substantial variability in reliability coefficients across studies and highlight a lack of evidence in specific populations, such as students, thereby limiting its generalizability [47]. These findings underscore the need for psychometric studies tailored to specific contexts and populations—such as nursing students—who face unique academic and clinical demands that increase their vulnerability to sleep disturbances.

In Latin America, validation studies of the PSQI and ESS among university students are limited, and even more scarce among nursing students. Given that psychometric properties can vary based on language, culture, and sample characteristics, it is essential to conduct context-specific analyses to confirm the construct validity, internal consistency, and practical utility of these scales [49]. Therefore, the objective of this study was to evaluate the psychometric properties of the PSQI and ESS in a sample of university nursing students. Specifically, we examined the structural validity, reliability, and convergent validity of both scales. Validating these instruments in nursing student populations is essential to ensure their adequacy, precision, and utility as assessment tools within health professional training programs. To the best of our knowledge, this is the first validation of these instruments in Colombian nursing students, addressing a critical gap in the assessment of sleep health within Latin American higher-education contexts. Furthermore, the resulting evidence may support the implementation of more holistic sleep assessments and inform the development of comprehensive well-being initiatives.

## Method

### Study design

This methodological study was guided by the COSMIN (Consensus-based Standards for the Selection of Health Measurement Instruments) initiative, which provides standardized recommendations for evaluating the psychometric properties of health-related measurement tools [50]. This study is part of a larger research project on sleep quality and academic performance among nursing students, and was conducted from the first academic term of 2023 through the first academic term of 2024.

The study was carried out in two phases. First, a cross-sectional phase, conducted between weeks 2 and 6 of the academic term—prior to the start of hospital practicums—to evaluate the structural validity, internal consistency, and convergent validity of the instruments; and second, a longitudinal phase, conducted between weeks 10 and 14, after the beginning of clinical practicums, in which students were invited to complete the same questionnaire again to assess test–retest reliability.

### Setting, sample, and sampling

This study was conducted at the Faculty of Health of a private university in Cali, Valle del Cauca, Colombia. The faculty offers full- and part-time undergraduate and postgraduate programs for the training of health professionals, including nurses.

The required sample size for conducting confirmatory factor analysis (CFA) of the PSQI and ESS, which together comprise 15 items/components, was estimated based on methodological recommendations. Specifically, it is advised to recruit 5 to 10 participants per item, or a minimum of 200 participants, to ensure stable and accurate parameter estimates [51].

A non-probability convenience sampling strategy was employed, targeting students enrolled in the undergraduate nursing program during the study period. The participants were approached in academic settings and informed about the purpose of the research. Those who voluntarily agreed to participate completed the instruments. The final sample included only students who met the predefined inclusion criteria.

For the longitudinal phase, all participants from the first phase were invited to take part in the second administration of the questionnaire to assess test–retest reliability.

### Inclusion and exclusion criteria

Students aged 18 years or older, who were enrolled in the undergraduate nursing program—at any stage of training (basic or professional cycle)—and who consented to participate in the study were included. Incomplete responses were excluded from the analysis.

## Measures

### Sociodemographic characteristics

Sociodemographic data were collected via a study-specific (ad hoc) questionnaire and included age, sex, marital status, geographic residence, and socioeconomic stratum. Age was recorded as a continuous variable (in completed years); sex was recorded as a dichotomous variable (men/women); and marital status as a categorical variable, which was later recoded into two groups: partnered (married or cohabiting) and not partnered (single, separated, widowed). Geographic residence was classified according to the participant’s usual place of residence and grouped as urban (within Cali) or nonurban (outside Cali). The socioeconomic stratum was self-reported based on the official classification assigned to the participant’s residence in Colombia (strata 1–6), which serves as an indicator of socioeconomic conditions—where stratum 1 represents the lowest level and stratum 6 the highest. For analysis, this variable was recoded into two categories: low and lower-middle (strata 1–3) and upper-middle and high (strata 4–6).

### Pittsburgh Sleep Quality Index (PSQI)

The PSQI is a 19-item self-report questionnaire that assesses global sleep quality over the past month [19]. The first four items collect information on sleep habits via open-ended responses. The remaining items address the presence of sleep disturbances and daytime difficulties, rated on a 4-point Likert scale according to the frequency or severity of the problem (0 = not during the past month; 1 = less than once a week; 2 = once or twice a week; 3 = three or more times a week). The instrument also includes a 5-item bed partner/roommate rating, which is not included in the scoring.

The PSQI items are grouped into seven components: subjective sleep quality, sleep latency, sleep duration, habitual sleep efficiency, sleep disturbances, use of sleeping medication, and daytime dysfunction. Each component is scored from 0 to 3, and their sum yields a global score ranging from 0 to 21. Higher scores indicate worse sleep quality, as established in the original validation study [19]. A global PSQI score > 5 indicates poor sleep quality, whereas a score ≤ 5 reflects good sleep quality [19]. For this study, we used the Spanish version of the PSQI validated in a Colombian population, which showed adequate internal consistency (Cronbach’s alpha = 0.78) [52].

### Epworth Sleepiness Scale (ESS)

The ESS is an 8-item self-report questionnaire designed to assess excessive daytime sleepiness [33]. The respondents rate their likelihood of dozing off or falling asleep in eight common daily situations: sitting and reading, watching television, sitting in a public place, riding as a passenger in a car, lying down to rest in the afternoon, sitting and talking to someone, sitting quietly after a meal, and being in traffic. Each item is rated on a 4-point Likert-type scale (0 = no chance, 1 = slight chance, 2 = moderate chance, 3 = high chance). The item scores are summed to produce a total score ranging from 0 to 24, with higher scores indicating greater levels of daytime sleepiness during common daily activities [33]. An ESS total score > 10 indicates excessive daytime sleepiness, whereas ≤ 10 reflects normal daytime sleepiness [33]. For this study, we used the Spanish version of the ESS validated in a Colombian population, which demonstrated adequate internal consistency (Cronbach’s alpha = 0.85) [53].

Although the Spanish versions of the PSQI and ESS were used, both scales were first subjected to qualitative evaluation by three expert judges with clinical and research experience—a psychologist, a nurse, and a physician—to verify the linguistic and contextual adequacy of the items. Additionally, a pilot test was conducted with 10 students who shared the characteristics of the target population to assess item clarity and comprehension. Based on the results of these evaluations, no modifications to the instruments were deemed necessary.

### Ethical considerations and procedure

This study received approval from the Institutional Ethics Committee of Universidad Libre, Cali, Colombia (record CEB-17-2022, April 4, 2022). All participants provided informed consent prior to enrollment. The investigators thoroughly explained the study objectives, clarified that participation entailed no foreseeable risks, and emphasized the right to withdraw at any time without penalty.

In the first phase, a total of 420 students who were regularly enrolled in the undergraduate nursing program were invited to participate. The invitation to participate was disseminated through institutional communication channels, and students were approached in various academic settings between February 2023 and May 2024. Although the invitation was distributed online, data collection was conducted in person. Participants completed the online form on-site at the university, with the support of trained research assistants who provided clarification when needed. This procedure ensured data accuracy and prevented entry errors while maintaining direct researcher accompaniment throughout the process.

In the second phase, participants who had taken part in the initial survey were recontacted through institutional channels and invited to complete the same online questionnaire again, following the same in-person procedure. Before participation, students were informed about the objectives of this phase and were asked to provide renewed informed consent prior to completing the form.

After completing the questionnaire, participants were provided with information about institutional psychological counseling and medical services, including contact details, to facilitate access to individual support through the university’s student welfare services, if needed.

### Data analysis

The first phase comprised descriptive statistics to characterize the sample. Quantitative variables are reported as the means and standard deviations, and categorical variables are reported as frequencies and percentages. Prior to the psychometric analyses, descriptive statistics of the PSQI and ESS were conducted as preliminary analyses. Global scores and component/item-level data were examined as continuous variables, while categorical distributions were analyzed according to cut-off criteria previously established in the literature [9, 19, 33, 34]. In this study, the seven PSQI components were treated as observed variables rather than individual items. The PSQI components represent the analytic unit proposed by the instrument’s author [19] and have consistently served as the basis for analysis in the literature [46].

Structural validity of the PSQI and ESS was assessed via confirmatory factor analysis (CFA). Given the ordinal nature of the observed variables, the weighted least squares mean- and variance-adjusted (WLSMV) estimator was employed [54]. For each scale, a theoretical unidimensional model was tested, positing that a single factor accounts for the variance in the observed indicators. Model fit was considered acceptable if the comparative fit index (CFI) and Tucker–Lewis index (TLI) were ≥ 0.95, and the root mean square error of approximation (RMSEA) was < 0.08 [55]. When the theoretical unidimensional models did not meet acceptable fit criteria, models were re-specified based on modification indices. Re-specifications were guided by theoretical and psychometric considerations, incorporating justified residual correlations between item pairs incrementally to improve model fit while maintaining model parsimony. The addition of residual correlations was discontinued once acceptable model fit was achieved.

To verify that the sample size was adequate to detect potential model misfit, a post hoc power analysis based on the RMSEA was conducted [56]. This analysis, performed with a significance level of α = 0.05, estimated the achieved power to detect meaningful deviations from close model fit (RMSEA ≥ 0.08) for each tested measurement model.

For measurement models that exhibited good data fit, we report standardized factor loadings and indicators of factor reliability (internal consistency). The reliability metrics included the ordinal alpha based on polychoric correlations [57], McDonald’s omega [58], composite reliability (CR) [59], and average variance extracted (AVE). Although the AVE has traditionally been employed as an indicator of convergent validity, contemporary literature supports its use as a measure of measurement precision [60]. Reliability values equal to or greater than 0.70 were considered indicative of adequate internal consistency [61].

For the subsample that completed the follow-up data collection, the intraclass correlation coefficient (ICC) was used to assess the test–retest reliability of the PSQI and ESS. A two-way random-effects model with absolute agreement was applied, following the classical classification proposed by Shrout and Fleiss [62]. Cut-off values for interpreting the ICC indicate that coefficients below 0.40 reflect poor reliability, values between 0.40 and 0.59 indicate fair reliability, values between 0.60 and 0.74 indicate good reliability, and values above 0.75 indicate excellent reliability [63].

Measurement error and agreement between test and retest scores for each instrument were further assessed using Bland–Altman dispersion plots. These plots display the differences between both administrations plotted against their means, providing a visual inspection of systematic bias and the 95% limits of agreement (LOA) that indicate the consistency of measurements across time [64].

Finally, as evidence of convergent validity based on an external criterion, we assessed the correlation between the PSQI and ESS measurement models that demonstrated acceptable fit. To this end, a structural model with correlated latent factors was estimated. This approach was selected because latent correlations provide a more precise estimate of the relationship between constructs by accounting for measurement error [65]. Model fit was evaluated via the same criteria applied to the individual measurement models. Correlation coefficients were interpreted as small (*r* ≈ 0.10), moderate (*r* ≈ 0.30), or large (*r* ≈ 0.50), based on established guidelines [66].

The data were analyzed in R (version 4.5.1) using the *lavaan* (version 0.6–16) [67] and *semTools* (version 0.5-6) [68] packages for CFA, the *irr* (version 0.84.1) [69] package to estimate the ICC for test–retest reliability, and the *semPower* (version 2.1.3) [56] package to perform the RMSEA-based power analysis.

## Results

### Sample characteristics

The study sample consisted of 368 participants, yielding a response rate of 87.6%. The sociodemographic features of the study participants are presented in Table 1. The mean age was 21.38 years. The majority of participants were women (77.72%) and reported being single (91.58%). With respect to geographic residence, 77.99% resided in urban areas of Cali. In terms of socioeconomic status, approximately four-fifths of the sample belonged to the lower socioeconomic strata (levels 1–3).

**Table 1:** Sociodemographic characteristics of the sample (*n* = 368)

Variables	M ± SD / *n* (%)
*Age*	21.38 ± 3.60
*Sex*	
Women	286 (77.72)
Men	82 (22.28)
Marital status	
Single	337 (91.58)
Married/Cohabiting	31 (8.42)
Geographic residence	
Urban (Cali)	287 (77.99)
Nonurban (Outside Cali)	81 (22.01)
Socioeconomic stratum	
1–3	292 (79.35)
4–6	76 (20.65)

Note. M: Mean, SD: Standard deviation

### Preliminary analyses of the instruments

Table 2 presents descriptive statistics for each PSQI component and ESS item. The mean scores for the PSQI components ranged from 0.81 (habitual sleep efficiency) to 1.70 (sleep duration), with skewness and kurtosis values falling within ± 1.5, except for the use of the sleeping medication component, which showed high skewness (3.09) and kurtosis (9.25). For the ESS, the item means ranged from 1.679 (watching television) to 0.628 (sitting and talking to someone), with all skewness and kurtosis values within ± 1.5 range.

**Table 2:** Descriptive statistics for PSQI components and ESS items (*n* = 368)

Components/ Items	M ± SD		Min	Max	Skewness	Kurtosis
**PSQI**						
Sleep quality	1.272 ± 1.076		0	3	0.023	-1.415
Sleep latency	1.397 ± 1.010		0	3	0.117	-1.073
Sleep duration	1.698 ± 1.051		0	3	-0.307	-1.097
Sleep efficiency	0.810 ± 0.943		0	3	1.034	0.141
Sleep disturbance	1.408 ± 0.645		0	3	0.409	0.009
Use of sleep medication	0.217 ± 0.615		0	3	3.090	9.251
Sleep daytime dysfunction	1.383 ± 0.970		0	3	-0.019	-1.029
**ESS**						
Sit/read	1.353 ± 0.960		0	3	0.060	-0.983
Watching tv	1.679 ± 1.028		0	3	-0.263	-1.062
Public place	0.984 ± 0.982		0	3	0.658	-0.641
Car passenger	1.630 ± 1.107		0	3	-1.175	-1.305
Lying down in the afternoon	0.929 ± 0.977		0	3	0.759	-0.486
Sit/talk to someone	0.628 ± 0.825		0	3	1.283	1.046
After lunch	1.370 ± 1.109		0	3	0.134	-1.331
Traffic	0.918 ± 1.041		0	3	0.820	-0.584

Note. M: Mean, SD: Standard deviation

**Fig. 1:**
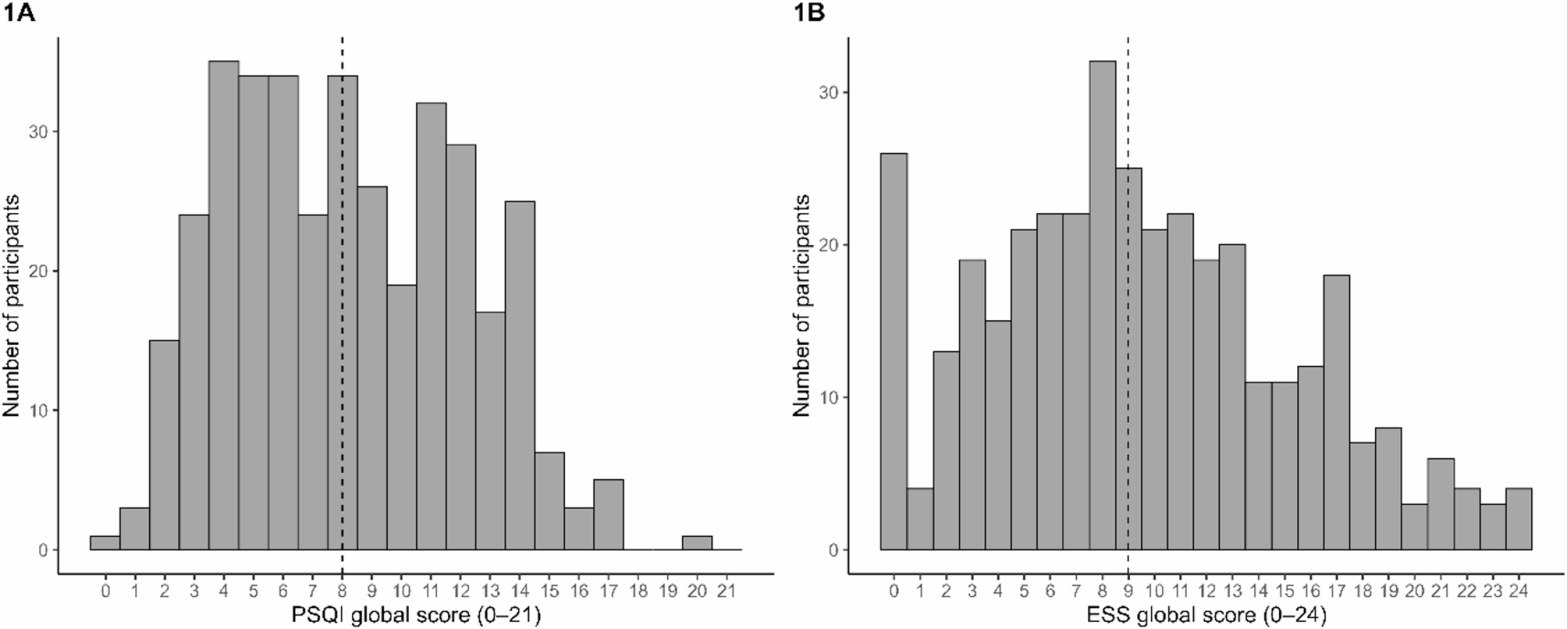
Frequency histograms of global scores for the PSQI (1 A) and ESS (1B) (*n* = 368)

Figure 1 presents the frequency histograms of the total scores for the PSQI and ESS. The mean global score for the PSQI was 8.19 (SD = 3.92; range: 0–20), while the mean score for the ESS was 9.49 (SD = 5.81; range: 0–24).

Table 3 summarizes the mean global scores and categorical distributions for the PSQI and ESS, using established cut-offs to classify sleep quality and daytime sleepiness. According to these criteria, 69.6% of students were classified as having poor sleep quality and 40.2% as experiencing excessive daytime sleepiness.

**Table 3:** Categorical distribution of PSQI and ESS global scores (*n* = 368)

Global scores/categorical distributions	M ± SD / *n* (%)
**PSQI**	
Global score	8.19 ± 3.92
Good sleep quality (≤ 5)	112 (30.4)
Poor sleep quality (> 5)	256 (69.6)
**ESS**	
Global score	9.49 ± 5.81
Normal daytime sleepiness (≤ 10)	220 (59.8)
Excessive daytime sleepiness (> 10)	148 (40.2)

Note. PSQI cut-off ≤ 5 = good sleep quality, >5 = poor sleep quality. ESS cut-off ≤ 10 = normal daytime sleepiness, >10 = excessive daytime sleepiness

### Confirmatory factor analyses

The results of the confirmatory factor analyses are presented in Table 4. The findings indicated that the theoretical unidimensional models for both the PSQI and ESS demonstrated acceptable CFI values; however, RMSEA values exceeded the recommended threshold (< 0.08), suggesting inadequate model fit. For the PSQI, the first re-specification suggested by the modification indices involved correlating the components of sleep quality and sleep duration. This re-specification did not improve model fit, as the RMSEA remained above the acceptable threshold (< 0.08). However, a second re-specification, which added a residual correlation between sleep duration and habitual sleep efficiency, resulted in acceptable fit indices (CFI and TLI > 0.95; RMSEA < 0.08). For the ESS, including a residual correlation between items 5 (lying down in the afternoon) and 6 (sitting and talking to someone) produced a model with acceptable fit (CFI = 0.997; RMSEA = 0.057). Details of the modification indices that guided these model re-specifications are provided in Supplementary Table S1.

**Table 4:** Model fit indices for the PSQI and ESS (*n* = 368)

Model	χ² (df)	CFI	TLI	RMSEA (90% CI)
**PSQI**				
Unidimensional (Theoretical)	110.731 (14)*	0.919	0.879	0.137 (0.114–0.162)
Respecified Unidimensional − 1 RC	79.044 (13)*	0.945	0.911	0.118 (0.093–0.143)
Respecified Unidimensional − 2 RC	36.527 (12)*	0.980	0.964	0.065 (0.048–0.073)
**ESS**				
Unidimensional (Theoretical)	157.188 (20)*	0.980	0.972	0.137 (0.117–0.157)
Respecified Unidimensional − 1 RC	41.667 (19)*	0.997	0.995	0.057 (0.033–0.081)

Note: χ² = chi-square test; df = degrees of freedom; CFI = Comparative Fit Index; TLI = Tucker–Lewis Index; RMSEA = Root Mean Square Error of Approximation; CI = Confidence interval; * = p value < 0.001; RC = Residual correlation

RMSEA-based post hoc power analyses indicated that, with α = 0.05 and *n* = 368, the achieved power to detect global misfit corresponding to RMSEA ≥ 0.08 was 0.967 for the PSQI (*df* = 12) and 0.996 for the ESS (*df* = 19). These findings confirm that the available sample size was sufficient to identify meaningful deviations from close model fit in both respecified measurement models.

**Fig. 2:**
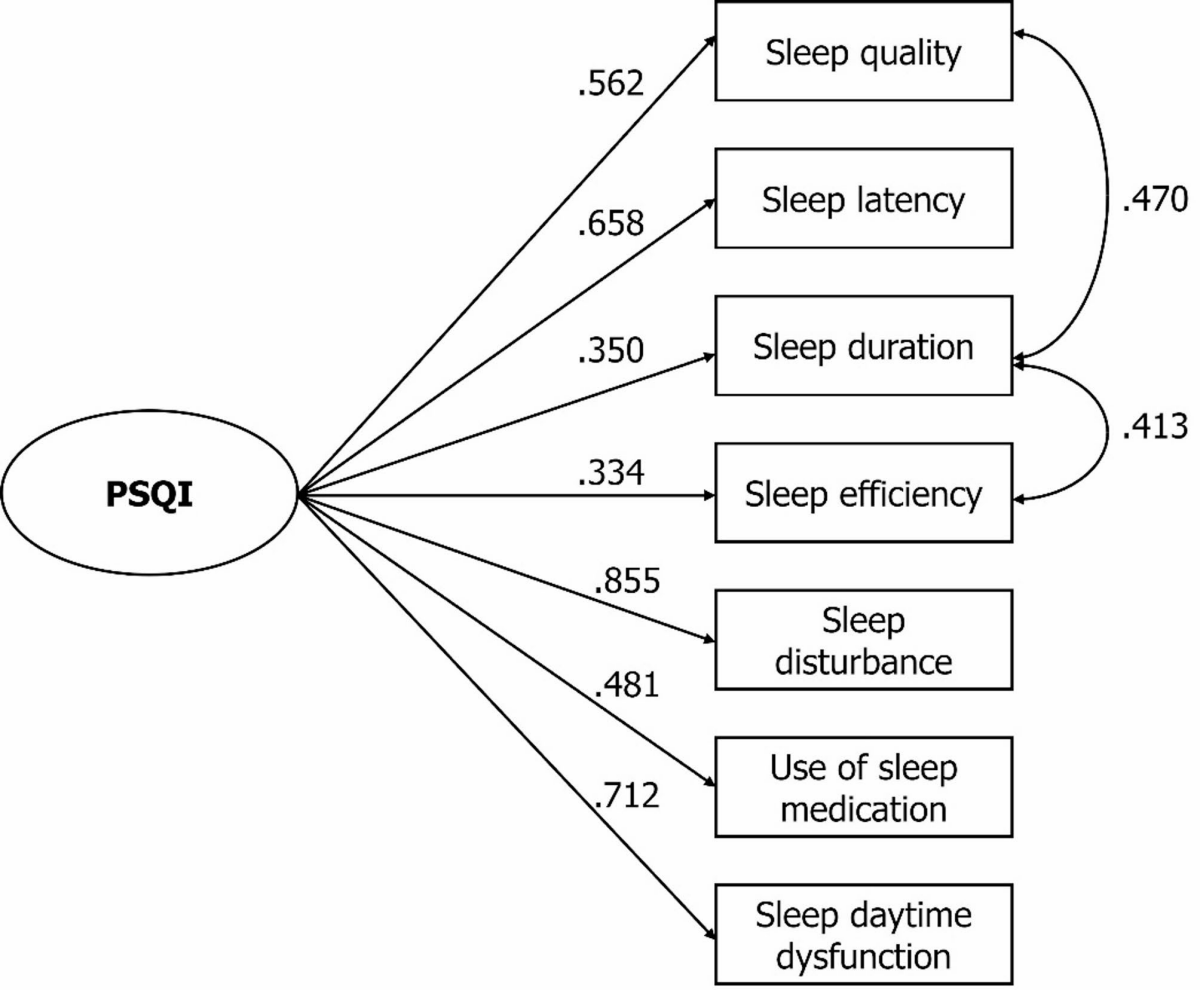
Identified structure and factor loadings of the PSQI (*n* = 368)

Figure 2 presents the standardized factor loadings for the PSQI. The loadings ranged from 0.334 to 0.855 (mean λ = 0.565). The sleep duration component showed residual correlations of 0.470 with subjective sleep quality and 0.413 with habitual sleep efficiency. All factor loadings and residual correlations were statistically significant (*p* < .001).

**Fig. 3:**
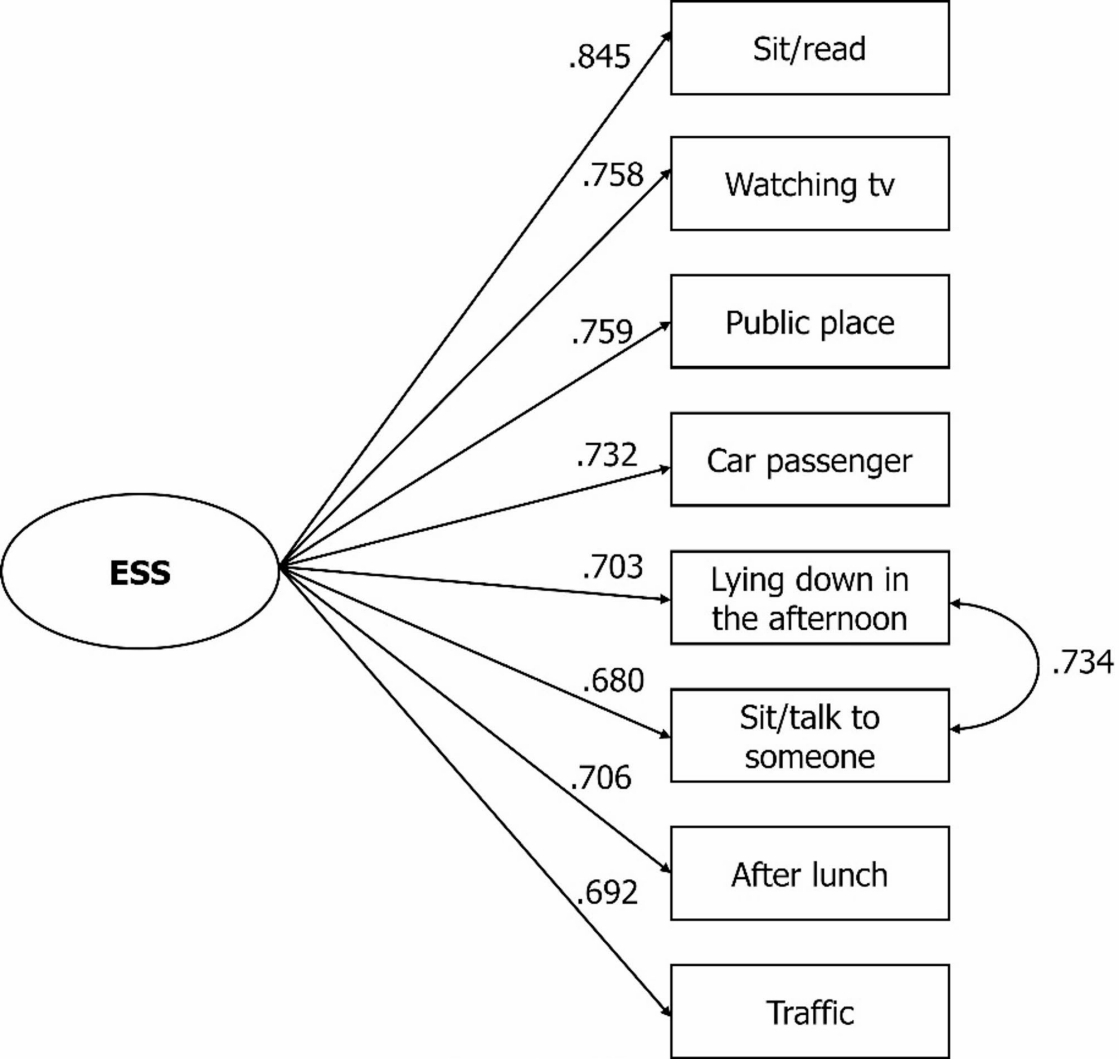
Identified structure and factor loadings of the ESS (*n* = 368)

Figure 3 displays the standardized factor loadings for the respecified unidimensional model of the ESS. Loadings ranged from 0.680 to 0.845 (mean λ = 0.734). The residual correlation between the items “lying down to rest in the afternoon” and “sitting and talking to someone” was 0.734. All factor loadings and residual correlations were statistically significant (*p* < .001).

### Reliability (Internal consistency)

Table 5 presents the reliability indices for the PSQI and ESS. For the PSQI, both ordinal alpha and composite reliability (CR) exceeded the acceptable threshold (> 0.70), indicating adequate internal consistency. However, McDonald’s omega (0.635) and the average variance extracted (AVE) fell below recommended cutoffs. In contrast, the ESS demonstrated excellent internal consistency (α = 0.901), along with high reliability as indicated by omega (0.858) and CR (0.904), both of which exceeded the minimum threshold. Additionally, the AVE for the ESS (0.541) surpassed the established cutoff, reflecting an adequate proportion of variance explained by the latent factor.

**Table 5:** Reliability indices for the PSQI and ESS (*n* = 368)

Scale	α	Ω	CR	AVE
PSQI	0.782	0.635	0.774	0.350
ESS	0.901	0.858	0.904	0.541

Note. α = ordinal alpha; Ω = McDonald’s omega; CR = composite reliability; AVE = average variance extracted

### Test–retest reliability

A total of 87 students from the original cohort completed the second assessment, forming the longitudinal subsample. The intraclass correlation coefficient (ICC) was used to assess the stability of the PSQI and ESS scores across time (Table 6). For the PSQI global score, the ICC was 0.464 (95% CI 0.282–0.614), indicating fair test–retest reliability. The ICCs for PSQI subcomponents ranged from 0.103 (95% CI − 0.073–0.283) for sleep duration to 0.452 (95% CI 0.269–0.604) for sleep disturbance. For the ESS, the global score yielded an ICC of 0.567 (95% CI 0.406–0.693), showing moderate reliability, with items ICCs ranging from 0.367 (95% CI 0.171–0.535) for “after lunch” to 0.539 (95% CI 0.372–0.672) for “watching tv”.

**Table 6:** Test-retest reliability for PSQI and ESS global score and subcomponents/items (*n* = 87)

Components/ Items	Test M ± SD	Retest M ± SD	ICC	95% CI
**PSQI global score**	7.172 ± 3.998	7.379 ± 3.773	0.464	0.282–0.614
Sleep quality	0.885 ± 0.993	1.080 ± 1.102	0.322	0.124–0.497
Sleep latency	1.575 ± 1.030	1.149 ± 0.922	0.420	0.213–0.586
Sleep duration	0.977 ± 1.034	1.667 ± 1.019	0.103	–0.073–0.283
Sleep efficiency	0.966 ± 1.094	0.736 ± 0.982	0.182	–0.024–0375
Sleep disturbance	1.368 ± 0.631	1.310 ± 0.616	0.452	0.269–0.604
Use of sleep medication	0.299 ± 0.749	0.218 ± 0.579	0.294	0.091–0.474
Sleep daytime dysfunction	1.103 ± 0.915	1.218 ± 0.945	0.216	0.010–0.407
**ESS global score**	8.333 ± 5.625	9.126 ± 5.906	0.567	0.406–0.693
Sit/read	1.092 ± 0.897	1.287 ± 0.926	0.463	0.283–0.612
Watching tv	1.655 ± 0.998	1.563 ± 0.949	0.539	0.372–0.672
Public place	0.805 ± 0.963	0.989 ± 1.006	0.472	0.294–0.620
Car passenger	1.552 ± 1.128	1.621 ± 1.123	0.528	0.358–0.664
Lying down in the afternoon	0.839 ± 1.033	0.885 ± 0.895	0.395	0.203–0.558
Sit/talk to someone	0.494 ± 0.791	0.667 ± 0.831	0.441	0.257–0.594
After lunch	1.138 ± 1.047	1.207 ± 1.143	0.367	0.171–0.535
Traffic	0.759 ± 0.988	0.908 ± 1.063	0.495	0.320–0.638

Note. M: Mean; SD: Standard deviation; ICC: Intraclass correlation coefficient; CI: Confidence interval

**Fig. 4:**
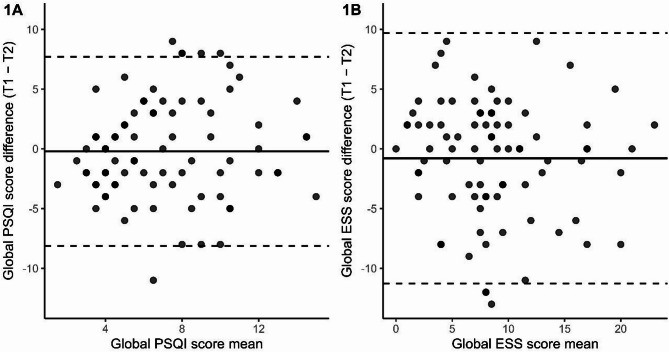
Bland –Altman dispersion plot of the mean PSQI and ESS global scores (*n* = 87)

Figure 4 depicts the Bland–Altman dispersion plots for the PSQI (1A) and ESS (1B). For the PSQI, the mean bias was − 0.21, with 95% limits of agreement (LOA) from − 8.12 to 7.71 and a standard error of measurement (SEM) of 2.86. For the ESS, the mean bias was − 0.79, with LOA between − 11.29 and 9.70 and a SEM of 3.79. In both instruments, the points were symmetrically distributed around the zero line, indicating no systematic bias and acceptable agreement between test and retest administrations.

### Convergent validity

The structural model assessing the correlation between the PSQI and ESS latent factors demonstrated acceptable fit: χ² (86) = 164.867, *p* < .001; CFI = 0.992; TLI = 0.991; RMSEA = 0.050, 90% CI [0.038, 0.061]. The latent correlation coefficient between the two scales was 0.721 (*p* < .001), indicating a strong association between sleep quality and daytime sleepiness.

## Discussion

This study aimed to evaluate the psychometric properties of the PSQI and ESS in a sample of university nursing students in Cali, Colombia. The findings indicate that both instruments exhibit adequate psychometric performance in this population, supporting their use in academic and similar non-clinical settings.

Preliminary analyses showed a mean PSQI score of 8.19 ± 3.92, with 69.6% of students classified as having poor sleep quality (PSQI > 5), and a mean ESS score of 9.49 ± 5.81, with 40.2% showing excessive daytime sleepiness (> 10). These proportions were higher than those reported in previous non-clinical samples [70, 71]. The cut-off points used in this study (PSQI > 5 and ESS > 10) are consistent with those most commonly applied in non-clinical and university populations, supported by evidence confirming their validity and adequate sensitivity to distinguish poor sleep quality and excessive daytime sleepiness [9, 34].

The confirmatory factor analyses of the PSQI and ESS revealed that, although the initial unidimensional models theoretically proposed by the original authors [19, 33] did not exhibit optimal fit in their initial specification, the proposed re-specifications—including residual correlations between conceptually related sleep components—substantially improved model fit in both cases. For the PSQI, residual correlations between sleep duration and subjective sleep quality, as well as between sleep duration and habitual sleep efficiency, supported a factor structure consistent with previous findings in adolescent populations [29, 32]. In the case of the ESS, the inclusion of a residual correlation between conceptually related items significantly enhanced model fit, in line with recent research [43, 45]. The literature has reported multiple factorial structures for the PSQI, including bifactor models [25, 32] and even trifactor models (Zhong et al., 2015), as well as bifactor structures for the ESS [37]. These structural variations may be influenced by cultural differences among samples, as sleep-related behaviors have been shown to be modulated by cultural and contextual factors [72].

The reliability analyses revealed differential performance between the instruments. The ESS demonstrated excellent internal consistency (ordinal α = 0.901) and high reliability, as indicated by omega (ω = 0.858) and composite reliability (CR = 0.904). The average variance extracted (AVE = 0.541) exceeded the recommended threshold, supporting an adequate proportion of variance explained by the latent factor. In contrast, the PSQI showed acceptable internal consistency (ordinal α = 0.782; CR = 0.774), but lower reliability values for omega (ω = 0.635) and AVE (0.350), both falling below commonly accepted cutoffs. These results suggest heterogeneity among components and potential residual multidimensionality.

These findings are consistent with the existing literature. In the case of the ESS, validation studies conducted in adult and university populations have reported acceptable to high reliability coefficients, supporting its unidimensional structure and use as a screening tool for daytime sleepiness [37, 41, 42, 45]. The reliability coefficients estimated in the present study (α, ω, and CR) fall at the upper end of those reported in the literature, indicating high internal consistency and supporting the applicability of the ESS in nursing student populations. In contrast, the PSQI yielded acceptable internal consistency (α = 0.782; CR = 0.774), but insufficient common factor reliability (ω = 0.635) and a low average variance extracted (AVE = 0.350). This pattern—α ≥ 0.70 with ω < 0.70 and AVE < 0.50—likely reflects the heterogeneous composition of the PSQI and suggests residual multidimensionality and/or local dependence among its components. Given that α can overestimate reliability in the presence of residual correlations, ω offers a more robust estimate of the shared variance attributed to the common factor. Therefore, the global PSQI score should be interpreted with caution. Consistent with previous research, unidimensional solutions often require local modifications or alternative bifactor/trifactor models [29, 31, 32]. In applied settings, it may be advisable to report and analyze component scores when examining specific associations and to complement them with convergent measures to strengthen interpretative validity. In contexts involving individual-level decision-making, the PSQI may benefit from being supplemented with additional measures or clinical evaluation criteria.

The test–retest analysis showed fair to moderate temporal stability for both instruments (PSQI ICC = 0.464; ESS ICC = 0.567). These values are slightly lower than those reported in recent studies, where ICC values ranged from 0.65 to 0.77 for the PSQI [24, 73] and from 0.78 to 0.81 for the ESS [40, 74]. Differences may be attributed to variations in the retest interval, the timing within the academic cycle, and contextual factors such as the start of hospital practicums, which can introduce natural fluctuations in sleep patterns rather than measurement inconsistency. Despite these factors, the absence of systematic bias in the Bland–Altman analysis supports the stability of both scales in this cohort, consistent with evidence from recent validation studies in non-clinical and clinical populations [18].

The convergent validity of the scales was supported by the structural model, which demonstrated acceptable fit (CFI = 0.992; TLI = 0.991; RMSEA = 0.050) and a strong latent correlation between the PSQI and ESS (*r* = .721; *p* < .001). This magnitude is consistent with previous evidence linking poorer sleep quality to greater daytime sleepiness across different age groups and settings [18, 29, 32]. Furthermore, validations of the ESS have consistently reported associations with sleep-related indicators, reinforcing its convergence with measures of sleep quality [37, 41, 42, 45]. Importantly, the observed correlation does not suggest conceptual redundancy. While the PSQI assesses multiple domains of sleep quality, the ESS specifically measures the propensity for daytime sleepiness. Thus, these instruments provide complementary information. Because the correlation was estimated at the latent level—controlling for measurement error—the evidence of convergence is robust. Nevertheless, given the documented factor heterogeneity of the PSQI, caution is warranted when inferring construct equivalence. In research contexts where greater specificity is required, it may be advisable to report and interpret component-level analyses [29, 32].

The interpretation of the results should consider both the study’s limitations and strengths. First, the single-center sample limit the external validity and generalizability of the findings. The PSQI and ESS rely on self-reported information, which may be subject to recall bias and response bias. These limitations could affect the accuracy of the reported sleep patterns and daytime sleepiness, and should be considered when interpreting the results.

Nonetheless, the adequate sample size and the use of multiple reliability indicators—ordinal alpha, McDonald’s omega, composite reliability (CR), and average variance extracted (AVE)—offer a robust assessment of internal consistency. Furthermore, to the best of our knowledge, this is the first study to jointly validate the PSQI and ESS in a sample of nursing students in Colombia, contributing valuable contextual evidence on the measurement of sleep quality and daytime sleepiness in this population.

Although the PSQI and ESS performed well in this sample, these findings should not be generalized to all academic or clinical training populations. Further validation in other health professional groups, such as physician interns or residents, is recommended.

Although test–retest evidence for the PSQI and ESS was included, these results should be interpreted with caution. First, the follow-up sample comprised 87 participants, which represents a relatively small subset of the total cross-sectional sample (*n* = 368) and may limit the generalizability of the temporal stability estimates. Second, the test–retest interval was approximately eight weeks, during which changes in sleep quality or daytime sleepiness could have occurred, potentially reflecting fluctuations in the constructs rather than measurement inconsistency.

Future studies should consider larger follow-up samples and employ longitudinal measurement invariance analyses across multiple time points. Sequentially testing configural, metric, scalar, and residual (strict) invariance would allow for a more rigorous evaluation of whether the PSQI and ESS retain stable measurement properties and interpretability over time.

Future research should prioritize multicenter validation studies that assess measurement invariance—configural, metric, and scalar—across relevant subgroups such as sex, academic semester, and mental health status. The incorporation of item response theory (IRT) analyses to evaluate individual item performance and assess differential item functioning (DIF) is also recommended. Additionally, strengthening convergent and discriminant validity through the integration of objective sleep measures (e.g., actigraphy, polysomnography) and clinical criteria is essential. It would also be pertinent to compare the performance of the PSQI using clinimetric approaches (e.g., risk scoring) with those under psychometric approaches (e.g., respecified factor models) in terms of their predictive validity for academic and clinical outcomes, as well as to determine optimal cut-points that best predict selected clinical or functional outcomes in student and trainee populations. Finally, future studies should examine the effectiveness of sleep hygiene interventions in university settings, using validated measures to assess impact on student well-being.

## Conclusion

The findings support the use of the PSQI and ESS as valid and reliable instruments for assessing sleep quality and daytime sleepiness among university nursing students. Their application facilitates a more comprehensive understanding of sleep health within academic settings. These results offer relevant evidence for both educational and clinical practice, suggesting that the use of these instruments may inform the development of institutional strategies aimed at promoting the well-being of health sciences students.

## Supplementary Information

Below is the link to the electronic supplementary material.

Supplementary Material 1

## Data Availability

The datasets generated during the current study are not publicly available due to protect the participants’ privacy but are available from the corresponding author on reasonable request.
