# Supplementary material for: Evaluating sleep quality and daytime sleepiness in nursing students: psychometric validation of the Pittsburgh Sleep Quality Index and the Epworth Sleepiness Scale
Source: BMC Nurs. 2025 Nov 25;24:1441. doi: 10.1186/s12912-025-04044-2 (PMC12648899; doi:10.1186/s12912-025-04044-2)
Supplement: Supplementary file 1 — Supplementary Material 1 [file 12912_2025_4044_MOESM1_ESM.docx]

**Supplementary material**

Evaluating Sleep Quality and Daytime Sleepiness in Nursing Students: Psychometric validation of the Pittsburgh Sleep Quality Index and the Epworth Sleepiness Scale

**Table S1. Modification indices supporting model re-specifications**

| **Residual correlation** | **MI** | **EPC** | ***sepc.all*** | **Decision** |
| --- | --- | --- | --- | --- |
| **PSQI** |  |  |  |  |
| Duration ~~ Efficiency | 31.54 | −0.32 | −0.43 | Retained (RC1) |
| Quality ~~ Duration | 29.34 | −0.32 | −0.52 | Retained (RC2) |
| Latency ~~ Disturbance | 17.48 | −0.27 | −0.58 | Not retained |
| Duration ~~ Disturbance | 15.94 | 0.29 | 0.58 | Not retained |
| Duration ~~ Use medication | 15.06 | 0.32 | 0.43 | Not retained |
| Latency ~~ Duration | 14.41 | 0.25 | 0.40 | Not retained |
| Efficiency ~~ Disturbance | 7.05 | 0.19 | 0.35 | Not retained |
| Quality ~~ Latency | 5.48 | 1.15 | 0.26 | Not retained |
| Latency ~~ Use medication | 5.38 | −0.19 | 0.26 | Not retained |
| Other pairs (MI < 5) | — | — | — | — |
| **ESS** |  |  |  |  |
| Lying down PM ~~ Talking | 115.50 | −0.43 | −1.96 | Retained (RC1) |
| Sit/read ~~ Watching TV | 20.71 | −0.19 | −0.48 | Not retained |
| Car passenger ~~ Talking | 20.58 | 0.27 | 0.83 | Not retained |
| Reading ~~ Lying down PM | 19.99 | 0.23 | 0.82 | Not retained |
| Public place ~~ Lying down PM | 16.64 | 0.22 | 0.66 | Not retained |
| Reading ~~ Talking | 16.47 | 0.23 | 0.84 | Not retained |
| Reading ~~ Public place | 15.75 | −0.17 | −0.42 | Not retained |
| Car passenger ~~ Traffic | 15.72 | −0.19 | −0.35 | Not retained |
| Public place ~~ Talking | 13.71 | 0.20 | 0.64 | Not retained |
| Car passenger ~~ Lying down PM | 11.46 | 0.18 | 0.53 | Not retained |
| Remaining pairs (MI < 10) | — | — | — | — |
| Notes. MI = Modification index; EPC = Expected parameter change; *sepc.all* = Standardized expected parameter change (all variables standardized); PSQI = Pittsburgh Sleep Quality Index; ESS = Epworth Sleepiness Scale; RC = Residual correlation. The residual correlations retained (two for PSQI and one for ESS) correspond to the model specifications under which acceptable fit indices were achieved (see Table 4 of the article). For the PSQI, were incorporated (duration–efficiency and quality–duration), resulting in the “Respecified Unidimensional – 2 RC” model. For the ESS, items 5 and 6 (lying down PM (in the afternoon) and sitting and talking to someone) was retained, producing the “Respecified Unidimensional – 1 RC” model. | | | | |
